# CONTRIBUTIONS OF HBCUS TO THE GERONTOLOGY FIELD: DISCUSSIONS OF RELIGION, HEALTH DETERMINANTS, AND BLACK AGING

**DOI:** 10.1093/geroni/igad104.0507

**Published:** 2023-12-21

**Authors:** Tamara Baker, Tiffany Washington

## Abstract

Health advancements have allowed older adults to a better quality of life. This however, is not equitably translated to the well-being of older Black adults. To bypass this narrative, actions are need to focus on restructuring fragmented systems that perpetuate health inequities and social injustices. This however cannot be the responsibility of one person, community, or institution, but rather a collective obligation of those willing to service the needs of all older adults. This allows us to not only acknowledge the scholarly work aimed at bettering the lives of our aging communities, but also recognizes the contributions of scholars affiliated with Historically Black Colleges and Universities (HBCUs), through research, policy, community interventions, and scholarly practices. To address this progressive movement, this collection of papers not only acknowledges the contributions of HBCUs to the gerontology discipline, but also to the scientific advancement focusing on the health and well-being of older Black adults. Speaker one will discuss the biopsychosocial determinants of cognitive function of older Black adults. Our second speaker will focus on religious coping and COVID-19 vaccine hesitancy among older Black individuals. Speaker three will highlight SDoH among older adults within Caribbean societies. The final speaker will discuss the impact and contributions of HBCU’s on an ’aging’ society through data analysis of demographic patterns and historical factors from an Afrocentric and Black perspectives. The discussant will tie together these themes and provide recommendations related to education, scholarly research ventures, particularly as it relates to health outcomes, equity, and collaborating with HBCUs. This is a HBCU Collaborative Interest Group Sponsored Symposium.

